# Transcriptomic profiling disclosed the role of DNA methylation and histone modifications in tumor-infiltrating myeloid-derived suppressor cell subsets in colorectal cancer

**DOI:** 10.1186/s13148-020-0808-9

**Published:** 2020-01-15

**Authors:** Varun Sasidharan Nair, Reem Saleh, Salman M. Toor, Rowaida Z. Taha, Ayman A. Ahmed, Mohamed A. Kurer, Khaled Murshed, Nehad M. Alajez, Mohamed Abu Nada, Eyad Elkord

**Affiliations:** 1 Cancer Research Center, Qatar Biomedical Research Institute (QBRI), Hamad Bin Khalifa University (HBKU), Qatar Foundation (QF), P.O. Box 34110, Doha, Qatar; 2 Department of Surgery, Hamad Medical Corporation, Doha, Qatar; 3 Department of Pathology, Hamad Medical Corporation, Doha, Qatar; 4 Biomedical Research Center, School of Science, Engineering and Environment, University of Salford, Manchester, UK

**Keywords:** Myeloid-derived suppressor cells, Colorectal cancer, Transcriptomic profile, DNA methylation, Histone modifications

## Abstract

**Background:**

Increased numbers of myeloid-derived suppressor cells (MDSCs) are positively correlated with poor prognosis and reduced survivals of cancer patients. They play central roles in tumor immune evasion and tumor metastasis. However, limited data are available on phenotypic/transcriptomic characteristics of the different MDSCs subsets in cancer. These cells include immature (I-MDSCs), monocytic (M-MDSCs), and polymorphonuclear/granulocytic (PMN-MDSCs).

**Methods:**

Phenotypic characterization of myeloid subsets from 27 colorectal cancer (CRC) patients was assessed by flow cytometric analyses. RNA-sequencing of sorted I-MDSCs, PMN-MDSCs, and antigen-presenting cells (APCs) was also performed.

**Results:**

We found that the levels of I-MDSCs and PMN-MDSCs were increased in tumor tissues (TT), compared with normal tissues (NT) in colorectal cancer. Our functional annotation analyses showed that genes associated with histone deacetylase (HDAC) activation- and DNA methylation-mediated transcriptional silencing were upregulated, and histone acetyl transferase (HAT)-related genes were downregulated in tumor-infiltrating I-MDSCs. Moreover, pathways implicated in cell trafficking and immune suppression, including Wnt, interleukin-6 (IL-6), and mitogen-activated protein kinase (MAPK) signaling, were upregulated in I-MDSCs. Notably, PMN-MDSCs showed downregulation in genes related to DNA methylation and HDAC binding. Using an ex vivo model, we found that inhibition of HDAC activation or neutralization of IL-6 in CRC tumor tissues downregulates the expression of genes associated with immunosuppression and myeloid cell chemotaxis, confirming the importance of HDAC activation and IL-6 signaling pathway in MDSC function and chemotaxis.

**Conclusions:**

This study provides novel insights into the epigenetic regulations and other molecular pathways in different myeloid cell subsets within the CRC tumor microenvironment (TME), giving opportunities to potential targets for therapeutic benefits.

## Background

Colorectal cancer (CRC) is the third most common cancer worldwide associated with high morbidity rates every year [1]. Inflammation is recognized as a key driver for CRC pathogenesis [2]. In inflammation-related cancers, myelopoiesis is disrupted and leads to accumulation of a heterogeneous population of myeloid cells, halted at varying stages of maturation/differentiation and with a potent immunosuppressive activity, referred to as myeloid-derived suppressor cells (MDSCs) [3, 4]. MDSCs express myeloid markers but lack expression of MHC class II molecule, HLA-DR, and are mainly divided into three phenotypically distinct subpopulations; CD33^+^HLA-DR^−/low^CD14^−^CD15^−^ early-stage, or immature MDSCs (e-MDSC/I-MDSC), which consist of immature myeloid progenitors; CD33^+^HLA-DR^−/low^CD14^+^CD15^−^ monocytic MDSCs (M-MDSCs), which represent suppressive monocytes; and CD33^+^HLA-DR^−/low^CD14^−^CD15^+^ polymorphonuclear or granulocytic MDSCs (PMN-MDSCs or G-MDSCs), which are phenotypically distinct from mature neutrophils and possess strong suppressive activity [3, 5–7].

MDSCs have been implicated in the pathogenesis of cancer [5, 8], where they act as potent suppressors of T cell-mediated responses against tumor cells within the tumor microenvironment (TME) [8] and lymphoid organs [9]. MDSCs enhance the progression of various types of tumors by promoting immune suppression, cancer-associated fibroblasts (CAF) activation, angiogenesis, and tumor growth and metastasis [10] via the expression of co-inhibitory receptors, such as PD-L1, and the release of a vast array of molecules, such as arginase-1, inducible nitric oxide synthase (iNOS), nitric oxide (NO), and reactive oxygen/nitrogen species (ROS/RNS) [11, 12]. Additionally, the number of circulating MDSCs has been positively correlated with poor prognosis and low survival rates in cancer patients, including those with CRC [13–15]. However, limited studies have explored the molecular/functional characteristics of different MDSC populations. In addition, the underlying mechanisms behind gene transcriptional regulation and signal transduction in these populations remain to be elucidated. Further insights into these mechanisms could provide potential candidates to target MDSC populations in cancer.

**Fig. 1:**
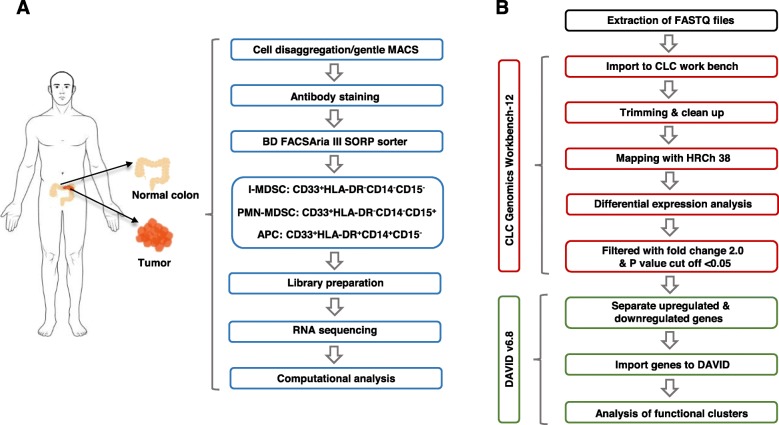
Schematic representation of the study design and analysis tools. The flowchart represents the work flow (**a**) and bioinformatics pipeline used in this study (**b**)

In this study, we performed flow cytometric analyses of MDSC subpopulations in the CRC TME. The overview of the study design and analyses pipelines is depicted in Fig. 1. We found that PMN-MDSCs and I-MDSCs were higher in CRC tumor tissues, compared with normal colon tissues. Moreover, the levels of these cells were higher than M-MDSCs in the TME. Additionally, we examined and transcriptomic profiling of I-MDSCs and PMN-MDSCs in TT versus adjacent NT using RNA-Seq analysis. We performed two-way comparisons of each population; firstly, we compared I-MDSCs or PMN-MDSCs in TT with corresponding NT, and secondly, we compared I-MDSCs or PMN-MDSCs in TT with APCs (the latter used as a control) in TT. Interestingly, our functional pathway analyses showed that epigenetic alterations, including DNA methylation (a common epigenetic mechanism by which gene transcription is repressed [16, 17]) and histone post-translational modifications (mediated either by histone deacetylases (HDACs) or histone acetyl transferases (HATs) differ from one myeloid cell population to another. Our RNA-Seq data showed that HDAC- and DNA methylation-related genes were significantly upregulated in tumor-infiltrating I-MDSCs, while HAT-related genes were downregulated. On the other hand, HDAC- and DNA methylation-related genes were significantly downregulated in tumor-infiltrating PMN-MDSCs. Moreover, Wnt, IL-6, MAPK, SNARE, JNK, and HIF-1 pathways were upregulated in tumor-infiltrating I-MDSCs, suggesting their potential involvement in cell differentiation and tumor progression. In this study, we also investigated the involvement of HDAC activation and IL-6 in regulating the suppressive function of MDSCs using an ex vivo model. Of note, further functional studies are warranted.

## Results

### Elevated levels of PMN-MDSCs and I-MDSCs in CRC tumor tissues

**Fig. 2:**
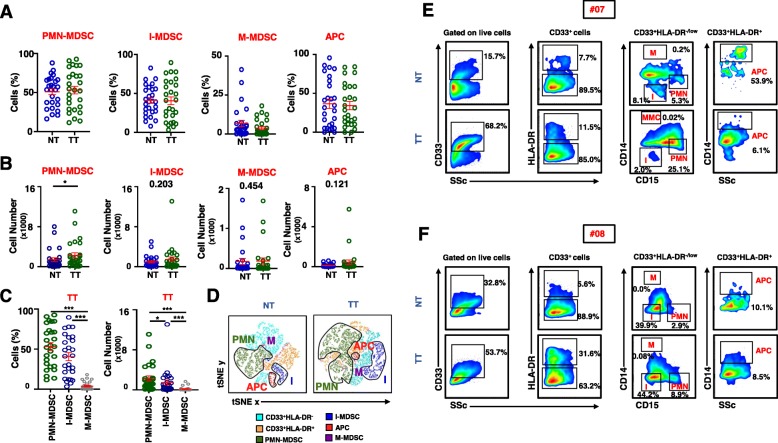
Comparison of myeloid populations (PMN-MDSCs, I-MDSCs, M-MDSCs, and APCs) in NT and TT of CRC patients. Cells isolated from NT and TT of 27 CRC patients were stained for myeloid cell markers and analyzed by flow cytometry. Scatter plots show the relative percentages of CD33^+^HLA-DR^−/low^CD14^−^CD15^+^ PMN-MDSCs, CD33^+^HLA-DR^−/low^CD14^−^CD15^−^ I-MDSCs, CD33^+^HLA-DR^−/low^CD14^+^CD15^−^ M-MDSCs, and CD33^+^HLA-DR^+^CD14^+^ APCs in NT and TT from 27 CRC patients (**a**). Scatter plots show differences in absolute numbers of PMN-MDSCs, I-MDSCs, M-MDSCs, and APCs in NT and TT from 27 CRC patients (**b**). Scatter plots show differences in relative percentages and absolute numbers of PMN-MDSCs, I-MDSCs, and M-MDSCs in TT from 27 CRC patients (**c**). Flow cytometric data were merged to create single t-distributed stochastic neighbor embedding (tSNE) maps to show PMN-MDSCs (denoted as PMN), I-MDSCs (denoted as I), M-MDSCs (denoted as M), and APCs in NT and TT (**d**). Representative flow cytometric plots show the gating strategy employed to define and sort PMN-MDSCs, I-MDSCs, M-MDSCs, and APCs from two CRC patients #07 (**e**) and #08 (**f**)

It has been reported that tumor-infiltrating PMN-MDSCs and I-MDSCs are expanded in colorectal tumor tissue, compared with normal colon tissue [13]. Here, we investigated the levels of PMN-MDSCs, I-MDSCs, M-MDSCs, and APCs in TT, compared with NT from the same CRC patients. The characteristic features of the study population are shown in Table 1. We determined the level of the different myeloid cell subsets in the TME (Fig. 2) and characterized their molecular/transcriptomic profiles. Cells were isolated from NT and TT of 27 CRC patients and flow cytometric analyses were performed for their phenotypical characterization. We did not find any significant differences in the relative percentages between TT and NT of CD33^+^HLA-DR^−/low^CD14^−^CD15^+^ PMN-MDSCs (53.1 ± 4.2 vs 51.0 ± 3.7), CD33^+^HLA-DR^−/low^CD14^−^CD15^−^ I-MDSCs (40.5 ± 5.0 vs 40.9 ± 3.7), CD33^+^HLA-DR^−^CD14^+^ M-MDSCs (3.8 ± 1.0 vs 6.8 ± 2.0), and CD33^+^HLA-DR^+^CD14^+^ APCs (34.3 ± 5.1 vs 36.6 ± 6.0) (Fig. 2a). Therefore, we compared the absolute numbers of these subsets between TT and NT (Fig. 2a). We found that the absolute numbers of PMN-MDSCs were significantly higher in TT compared to NT (2272.0 ± 526.0 vs 1384.0 ± 383.5, Fig. 2b). However, although the absolute numbers of I-MDSCs were higher in TT compared to NT, the data did not reach statistical significance (1467.0 ± 490.7 vs 975.8 ± 230.9, Fig. 2b). Moreover, there were no significant differences in the absolute numbers of M-MDSCs and APCs between TT and NT (Fig. 2b). In addition, we compared the overall levels of PMN-MDSCs, I-MDSCs, and M-MDSCs in TT and found that the relative percentages and absolute numbers of PMN-MDSCs, followed by I-MDSCs were significantly higher than M-MDSCs in the CRC TME (Fig. 2c). Visualization of the different myeloid cell subsets in NT and TT is depicted in Fig. 2d. We generated t-distributed stochastic neighbor embedding (tSNE) plots for markers of myeloid cell subsets and confirmed that PMN-MDSCs and I-MDSCs were higher in TT, while APCs showed similar levels and M-MDSCs were present at very low levels in NT and TT.

**Table 1:** Characteristic features of study populations

	CRC patients
Number	27 (2)*
Age	59 (31–96)^†^
Gender (male:female)	20:7
TNM stage	
I	2
II	8 (1)*
III	14 (1)*
IV	3
Histological grade	
G2—moderately differentiated	All samples

*CRC* colorectal cancer

*Samples used for transcriptomic profiling of tumor-infiltrating

myeloid cells

^†^Data shown represent median (range)

Next, we selected six CRC patients to sort different myeloid cell subpopulations (I-MDSCs, PMN-MDSCs, and APCs) from TT and NT and analyze their transcriptional profiling using RNA-Seq. However, the library preparation and subsequent RNA-sequencing were succeeded from two patients only (#07 and #08). Gating strategy for sorting these subpopulations from the two patients is shown in Fig. 2e and f.

### Genes associated with HDAC activation, DNA methylation, and IL-6 signaling pathway are upregulated in tumor-infiltrating I-MDSCs

**Fig. 3:**
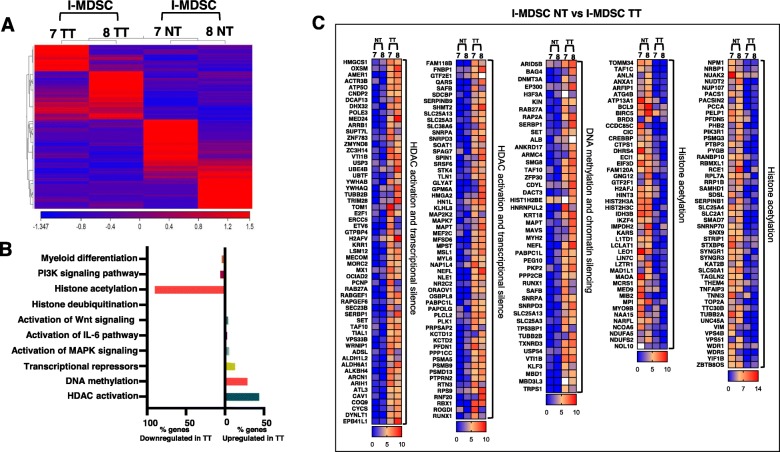
Differential gene expression of I-MDSCs in CRC microenvironment. Hierarchical clustering of I-MDSCs from two TT and NT (from patients #07 and 08) on differentially expressed RNA transcripts from RNA-Seq data. Each column represents a sample and each row represents a transcript. Expression level of each gene in a single sample is depicted according to color scale (**a**). Functional categorization of both upregulated and downregulated top significantly affected transcripts (*P* value < 0.05) from CLC analysis were analyzed through DAVID separately. The total number of genes come under both downregulated and upregulated categories was used to calculate the percentage of genes come under individual functional category. The bar diagram shows the percentage of genes present in each functional category (**b**). Heat maps show the log2 of transcript per million (TPM) representing fold change relative to the mean expression of HDAC activation and transcriptional silence, DNA methylation, and chromatin silencing and histone acetylation in TT, compared with NT (**c**)

**Fig. 4:**
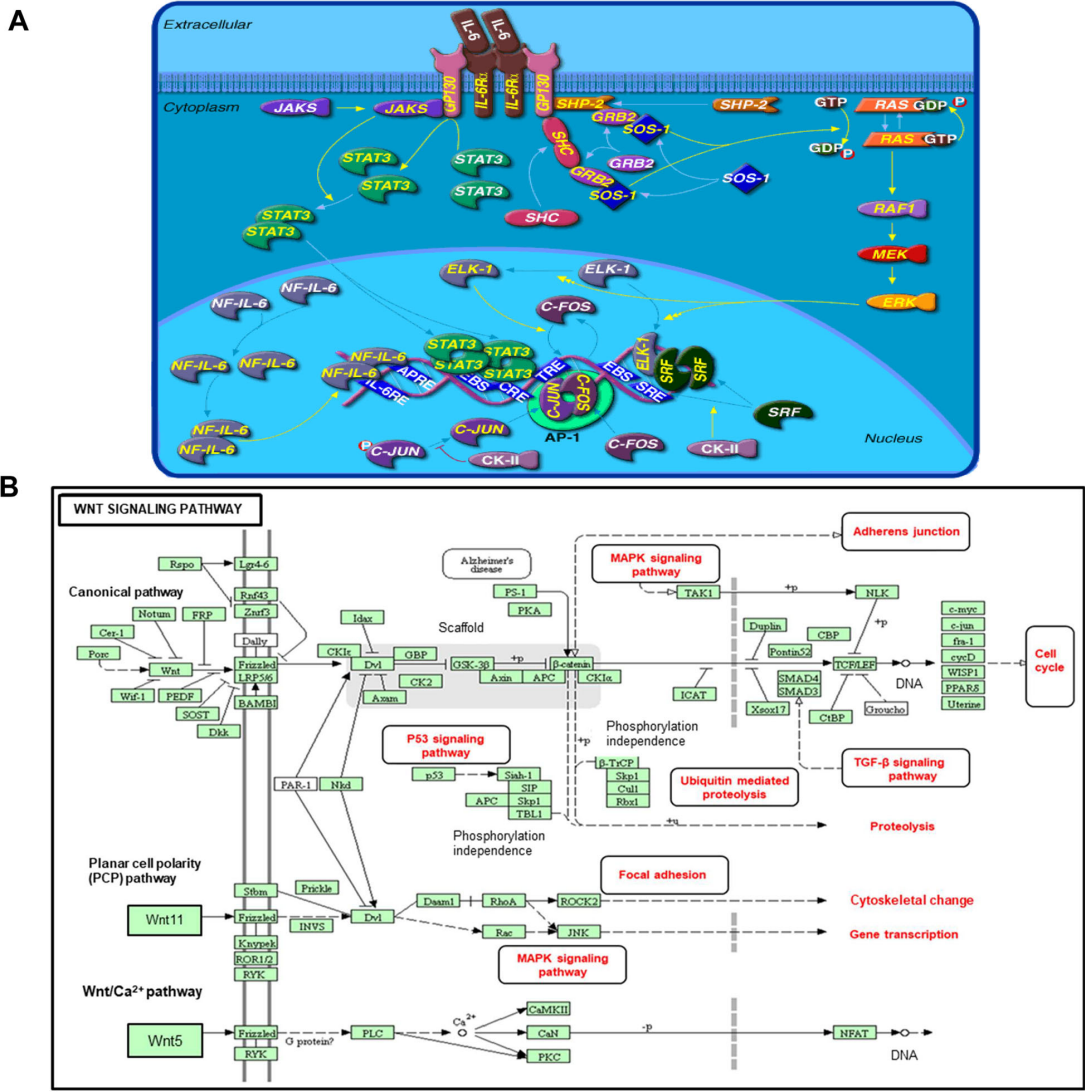
Functional network analysis of I-MDSCs in CRC microenvironment. The significantly upregulated genes in TT, compared with NT from 2 patients were uploaded in DAVID to identify the biological pathways. BioCarta pathway of IL-6 signaling pathway is identified in the enrichment analysis with significant enrichment of upregulated genes with *P* < 0.05 (**a**). WNT signaling is the top KEGG pathway regulated in I-MDSCs, compared with APCs within the TME (**b**). The black ovals highlight the KEGG identified functional pathways that are regulated by I-MDSCs, within the TME. The red highlights without boxes show the functional consequences of related genes

A study showed that I-MDSCs can migrate into the TME and differentiate into the highly immune suppressive tumor-associated macrophages (TAMs) and contribute to tumor progression by inhibiting the adaptive anti-tumor immune response and enhancing tumor growth/metastasis [18]. Here, we analyzed the differential gene expression in I-MDSCs isolated from TT, compared with those isolated from NT. The hierarchal clustering of differentially expressed transcripts showed a distinct cluster of I-MDSCs in NT and TT (Fig. 3a and Additional file 2: Table S1). Seven hundred ninety-eight upregulated and 637 downregulated transcripts were identified in tumor-infiltrating I-MDSCs, compared with those found in NT (with a fold of change > 2, *P* value cutoff < 0.05). Functional annotation analyses showed that upregulated genes are involved in histone deubiquitination (6 genes), activation of Wnt signaling pathway (4 genes), activation of IL-6 pathway (3 genes), activation of MAPK signaling (5 genes), transcriptional repressors (13 genes), DNA methylation (29 genes), and HDAC activation (44 genes) (Fig. 3b, c and Additional file 3: Table S2). Interestingly, epigenetic alterations such as post-translational histone modifications were predominant in I-MDSCs (Fig. 3b, c). We found that 148 of upregulated genes in tumor-infiltrating I-MDSCs are involved in HDAC activation and 50 upregulated genes are associated with DNA methylation (Fig. 3b, c). Studies showed that activation of IL-6 [19] and Wnt signaling pathways [20] can promote the trafficking of MDSCs to the TME. In line with this, our data showed that 10 genes associated with IL-6 pathway and 12 genes related to Wnt signaling are significantly upregulated in tumor-infiltrating I-MDSCs, compared with those found in NT (Fig. 3b. c). This implies a potential role of IL-6 and Wnt signaling pathways (Fig. 4a, b) in the biology of tumor-infiltrating I-MDSCs. Notably, 112 genes related to HAT activity and 12 genes related to myeloid differentiation were significantly downregulated in tumor-infiltrating I-MDSCs, compared with I-MDSCs in NT (Fig. 3b, c).

Collectively, our data show that the transcriptional profile of tumor-infiltrating I-MDSCs could be altered by histone post-translational modifications induced by HDAC activation, and/or DNA methylation. In addition, we speculate that I-MDSCs promote tumorigenesis by activating signaling pathways, such as Wnt and IL-6 pathways, which in turn enhance MDSCs recruitment to the TME and support MDSCs suppressive function.

### Genes associated with chemotaxis, cell migration, and anti-apoptosis are upregulated in tumor-infiltrating PMN-MDSCs

**Fig. 5:**
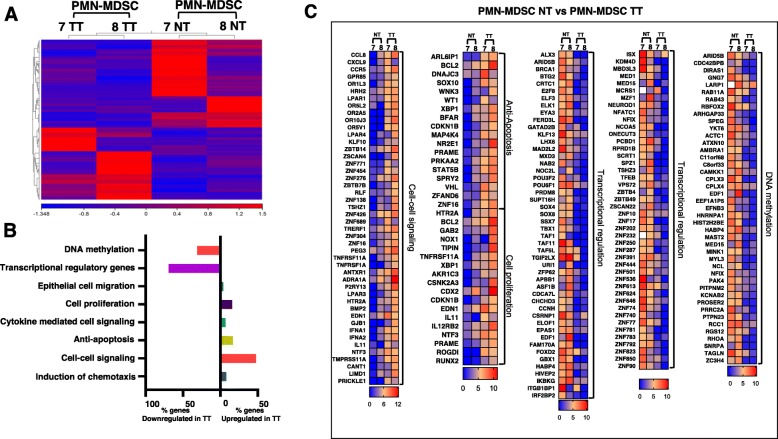
Differential gene expression of PMN-MDSCs in CRC microenvironment. Hierarchical clustering of PMN-MDSCs from two TT and NT (from patients #07 and #08) on differentially expressed RNA transcripts from RNA-Seq data. Each column represents a sample and each row represents a transcript. Expression level of each gene in a single sample is depicted according to color scale (**a**). Functional categorization of both upregulated and downregulated top significantly affected transcripts (*P* value < 0.05) from CLC analysis were analyzed through DAVID separately. The total number of genes come under both downregulated and upregulated categories were used to calculate the percentage of genes come under individual functional category. The bar diagram shows the percentage of genes present in each functional category (**b**). Heat maps show the TPM representing fold change relative to the mean expression of cell-cell signaling, anti-apoptosis, cell proliferation, transcriptional regulation, and DNA demethylation in TT, compared with NT (**c**)

Next, we investigated the differential gene expression in tumor-infiltrating PMN-MDSCs. It has been reported that PMN-MDSCs expansion within the CRC tissue is positively correlated with advanced disease stages and high histological grades, implying a role for PMN-MDSCs in CRC progression [13]. The hierarchal clustering of differentially expressed transcripts showed a distinct cluster of PMN-MDSCs isolated from NT and TT (Fig. 5a and Additional file 2: Table S1). Five hundred seventy-six upregulated and 785 downregulated transcripts were identified in tumor-infiltrating PMN-MDSCs, compared with those found in NT (with a fold of change > 2 and *P* value cutoff < 0.05). Within the upregulated transcripts of PMN-MDSCs, 5 genes were related to chemotaxis induction, 60 genes were related to cell-cell signaling, 22 genes were related to anti-apoptosis, 9 genes were related to cytokine-mediated signaling, 20 genes were related to proliferation and 4 genes were related to epithelial migration (Fig. 5b, c and Additional file 3: Table S2). Interestingly, 98 genes related to transcriptional regulation and 45 genes related to DNA methylation were significantly downregulated in tumor-infiltrating PMN-MDSCs, compared with those found in NT (Fig. 5b, c). In addition, our functional network analysis confirms that cell migration-related genes were upregulated in the PMN-MDSCs subpopulation found within the CRC microenvironment (Additional file 1: Figure S1).

### Transcriptional regulation in tumor-infiltrating I-MDSCs and PMN-MDSCs is mediated via distinct epigenetic mechanisms

**Fig. 6:**
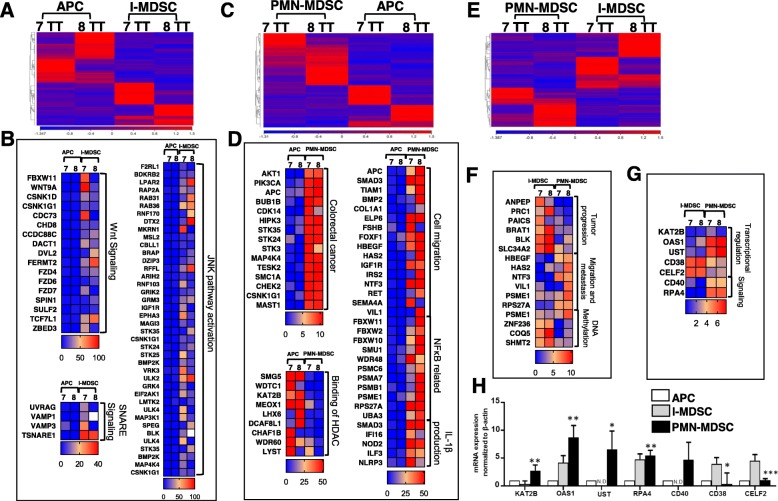
Differential gene expression and functional network analyses of APCs, PMN-MDSCs, and I-MDSCs in CRC microenvironment. Hierarchical clustering of APCs and I-MDSCs from two TTs (patients #07 and #08) on differentially expressed RNA transcripts from RNA-Seq data (**a**). Heat maps show the TPM representing fold change relative to the mean expression of WNT signaling, SNARE signaling, and JNK pathway activation in I-MDSCs, compared with APCs (**b**). Hierarchical clustering of PMN-MDSCs and I-MDSCs from two TTs on differentially expressed RNA transcripts from RNA-Seq data (**c**). Heat maps show the TPM representing fold change relative to the mean expression of colorectal cancer-, binding of HDAC-, cell migration-, NFκB-, and IL-1β production-related genes in PMN-MDSCs, compared with APCs (**d**). Hierarchical clustering of PMN-MDSCs and I-MDSCs from two TTs on differentially expressed RNA transcripts from RNA-Seq data (**e**). Heat map shows the TPM representing fold change relative to the mean expression of tumor progression-, migration and metastasis-, and DNA methylation-related genes in PMN-MDSCs, compared with I-MDSCs (**f**). Heat map shows the TPM representing fold change relative to the mean expression of genes associated with transcriptional regulation and signal transduction genes in PMN-MDSCs, compared with I-MDSCs (**g**). The mRNA expression levels for selected genes in tumor-infiltrating sorted myeloid cells, PMN-MDSCs vs. APCs were validated by RT-PCR (**h**). The relative gene expression was normalized to β-actin. Results obtained from six CRC patients, #05, #07, #08, #09, #44, and #53, and expressed as mean ± SEM. N.D. not detected

Next, we investigated the differential expression of transcripts in tumor-infiltrating I-MDSCs, compared to APCs. The hierarchal clustering of gene transcripts is depicted in Fig. 6a and Additional file 2: Table S1. We found 1802 transcripts that were differentially expressed in I-MDSCs. Out of 1802 transcripts, 1098 were significantly upregulated and 704 were downregulated in I-MDSCs (with a fold of change > 2 and *P* value cutoff < 0.05). Based on the network and functional annotation analyses, 17 genes from Wnt signaling, 41 genes from JNK pathway and 4 genes from SNARE complex activation were upregulated in tumor-infiltrating I-MDSCs (Fig. 6b and Additional file 3: Table S2).

We also investigated the differences in the transcriptional profile between tumor-infiltrating PMN-MDSCs and APCs. The hierarchal clustering of PMN-MDSCs and APCs is shown in Fig. 6c and Additional file 2: Table S1. We found 1803 genes that were distinct in PMN-MDSCs, compared with APCs. Out of 1803, 1098 genes were significantly upregulated and 705 were downregulated in tumor-infiltrating PMN-MDSCs, compared with APCs (with a fold of change > 2 and *P* value cutoff < 0.05). Interestingly, 15 genes related to CRC progression, 19 genes related to cell migration and 17 genes related to NF-kB/IL-1β-mediated recruitment of MDSCs were upregulated in tumor-infiltrating PMN-MDSCs (Fig. 6d and Additional file 3: Table S2). Moreover, 13 genes related to HDAC binding and 17 genes related to DNA methylation were significantly downregulated in tumor-infiltrating PMN-MDSCs (Fig. 6d). Together, these results suggest that upregulation of genes involved in cell migration and MDSCs recruitment in tumor-infiltrating PMN-MDSCs may occur as a result of HDAC inactivation and DNA demethylation.

Additionally, we compared the transcription profile between tumor-infiltrating PMN-MDSCs and I-MDSCs. The hierarchal clustering is shown in Fig. 6e and Additional file 2: Table S1. We found that 1395 genes were significantly distinct in tumor-infiltrating PMN-MDSCs. Out of 1395, 633 genes were significantly upregulated and 762 were downregulated in tumor-infiltrating PMN-MDSCs. Functional annotation analyses showed that 6 genes related to tumor progression and 6 genes related to migration were significantly upregulated, while DNA methylation-related genes were significantly downregulated in PMN-MDSCs, compared with I-MDSCs (Fig. 6f and Additional file 3: Table S2). These data are consistent with our findings above (PMN-MDSCs TT vs. NT, Fig. 5; I-MDSCs TT vs. NT, Fig. 3). In addition, we found that genes associated with transcriptional regulation (KAT2B, OAS1, UST, CD38, and CELF2) and signal transduction (CD40 and RPA4) were differentially expressed in PMN-MDSCs and I-MDSCs (Fig. 6g). We validated the expression of the genes above in tumor-infiltrating PMN-MDSCs, I-MDSCs, and APCs (the latter used as a control) by RT-PCR. We found that mRNA expression levels in PMN-MDSCs vs. I-MDSCs (Fig. 6h) were consistent with RNA-Seq data (Fig. 6g). Statistical significance was obtained by comparing the mRNA expression levels in PMN-MDSCs vs. I-MDSCs (Fig. 6h). Interestingly, our functional network analysis shows that HIF-1 pathway was active in tumor-infiltrating I-MDSCs (Additional file 1: Figure S2). These data are consistent with previous reports demonstrating that HIF-1α is essential for the infiltration and differentiation of myeloid cells within the TME [21, 22]. Additionally, we validated the functional clusters for patients #07 and #08 in four independent data sets (Additional file 1: Figure S3). Adhesion molecules, such as integrins, have been implicated in cancer progression [23]. It was reported that recruitment of neutrophils (a granulocytic subset that display similar phenotypic/functional characteristics as PMN-MDSCs) to tumor sites is associated with increased expression of CD11a integrin on neutrophils (also known as LFA-1) [24]. In this study, our data from RNA-Seq analysis showed that ITGAL gene encoding CD11a integrin is upregulated in tumor-infiltrating PMN-MDSCs, compared with I-MDSCs (Additional file 1: Figure S4A). We validated this finding by flow cytometric analysis and showed that CD11a is highly expressed on tumor-infiltrating PMN-MDSCs, compared with I-MDSCs (Additional file 1: Figure S4B).

### Genes associated with immunosuppression and myeloid cell recruitment in CRC tumor tissue are downregulated upon HDAC or IL-6 inhibition

**Fig. 7:**
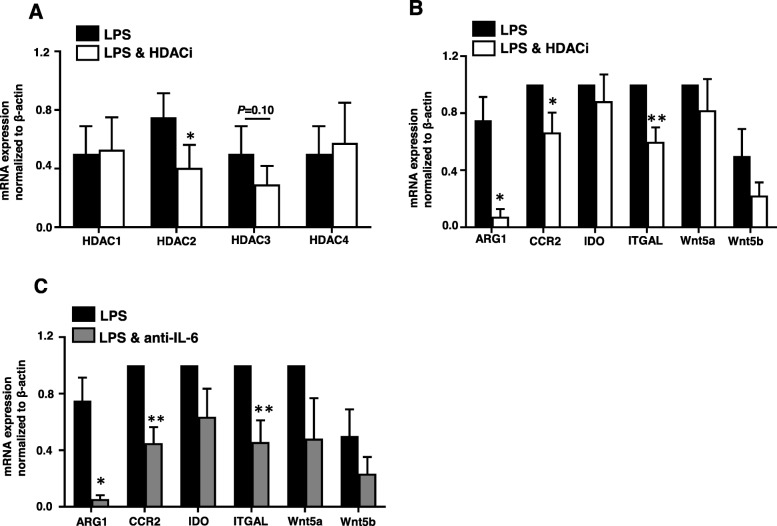
Effect of HDAC or IL-6 inhibition on genes associated with immunosuppression and myeloid cell recruitment in CRC tumor tissues. Cells isolated from CRC tissue were cultured in the presence of LPS and HDAC inhibitor (100 nM) or anti-IL-6 mAb (2 μg/ml). The relative mRNA expression levels for HDAC1, HDAC2, HDAC3, HDAC4 (**a**), and ARG1, CCR2, IDO, ITGAL, Wnt5a, and Wnt5b were determined in the absence or presence of HDAC inhibitor (**b**). The relative mRNA expression levels for ARG1, CCR2, IDO, ITGAL, Wnt5a, and Wnt5b were determined in the absence or presence of anti-IL-6 mAb (**c**). The relative gene expression was normalized to β-actin. Results obtained from four CRC patients, #26, #56, #60 and #63, and expressed as mean ± SEM.

Our results from RNA-Seq analysis suggested that genes associated with HDAC activation and IL-6 signaling pathway were upregulated in tumor-infiltrating I-MDSCs, compared to those isolated from NT (Figs. 3 and 4). We utilized an explant culture model and cultured cells isolated from CRC tumor tissues to examine the effect of HDAC inhibition and IL-6 neutralization on the expression of genes related to MDSCs function or recruitment. We examined the expression of four HDAC genes in addition to ARG1 (encodes arginase 1 enzyme, which synthesizes nitric oxide), ITGAL (CD11a, which could be an important mediator for PMN-MDSCs migration into tumor tissue), and IDO (encodes indoleamine 2,3-dioxygenase enzyme, which possess an immunosuppressive function). We also examined the expression of Wnt5a and Wnt5b as RNA-Seq data indicated the potential importance of Wnt signaling in I-MDSCs biology/function. Briefly, cells from CRC tumor tissue were cultured in the presence of LPS pre-treated or untreated with HDAC inhibitor or anti-IL-6. HDAC inhibitor and anti-IL-6 mAb were added to cell culture every other day. Of note, LPS was used as a mitogen to retrieve/enhance the survival of myeloid cells and induce their activation [25]. On day 5, gene expression analysis was performed using RT-PCR. The addition of HDAC inhibitor did not alter the expression level of HDAC1 and HDAC4 mRNA. However, the expression of HDAC2 and HDAC3 were reduced, compared to LPS alone (Fig. 7a). HDAC inhibitor significantly reduced the expression of ARG1, CCR2 (monocyte chemokine receptor), and ITGAL, compared with LPS alone (Fig. 7b). HDAC inhibition had no effects on the expression level of IDO, Wnt5a, and Wnt5b (Fig. 7b). The neutralization of IL-6 significantly reduced the mRNA levels of ARG1, CCR2, and ITGAL (Fig. 7c). IL-6 neutralization, however, did no not alter the expression level of IDO, Wnt5a, and Wnt5b (Fig. 7c). Collectively, these results indicate the importance of HDAC activation and IL-6 signaling pathways in regulating immunosuppression and myeloid cell recruitment to CRC tumor tissues.

## Discussion

Tumor-infiltrating MDSCs are key players, which negatively modulate the anti-tumor immune response, and promote angiogenesis and tumor growth/metastasis [26, 27]. Elevated levels of circulating and tumor-infiltrating PMN-MDSCs and I-MDSCs have been detected in CRC patients [13, 28]. Additionally, the expansion of tumor-infiltrating PMN-MDSCs in CRC patients has been positively correlated with advanced disease stages and high histological grades [13]. However, the phenotypic and molecular characteristics of these MDSCs subsets and their role in CRC progression remain elusive. Therefore, we investigated the transcriptional profiles and functional characteristics of I-MDSCs and PMN-MDSCs within the CRC microenvironment.

The phenotypic and functional characterization of MDSCs is widely known to be challenging due to the heterogeneous nature of these cells [27]. In this study, we utilized myeloid cell markers to identify the different subsets of MDSCs, to calculate their levels in colorectal tissues as previously described [13]. We found that the numbers of PMN-MDSCs and I-MDSCs are higher in colorectal tissues, compared to adjacent normal colorectal tissues. This falls in agreement with other reports demonstrating the expansion of different MDSCs subsets in the TME of several malignancies [29, 30].

In this study, transcriptional profiling analyses of I-MDSCs and PMN-MDSCs revealed novel mechanisms/signaling pathways, which may govern the MDSCs-mediated immune suppression and tumor progression in CRC. Our canonical and functional pathway analyses of I-MDSCs showed that Wnt, IL-6, and MAPK signaling pathways are significantly upregulated in TT, compared with NT, suggesting a role of these pathways in tumor-infiltrating I-MDSCs within the CRC microenvironment. The involvement of Wnt signaling in CRC pathogenesis and progression has been previously established [31–33]. Using a co-culture system with breast cancer cell lines, Pukrop et al. showed that Wnt signaling is crucial for macrophage-mediated tumor invasiveness and tumor cell migration via the production of matrix metalloproteinases (MMPs) [34]. Wnt pathway has been shown to antagonize the recruitment and differentiation of MDSCs in tumor sites [35]. However, our data suggest a potential role of Wnt signaling in one MDSCs subset (I-MDSCs) from CRC tissues. This discrepancy could be due to the heterogeneity of MDSCs and suggests that signaling pathways could impact subsets of MDSCs differentially. The importance of IL-6 signaling pathway in MDSCs recruitment and immune suppression has been previously demonstrated [30]. Using breast cancer in vitro and in vivo models, Jiang et al. reported that exogenous IL-6 augments the accumulation of MDSCs and enhances T cell suppression by inhibiting SOCS3 pathway [19]. In another study, it was demonstrated that IL-6 upregulates the expression of indoleamine-pyrrole 2,3-dioxygenase (IDO) enzyme via NF-κB activation, thereby enhancing the immunosuppressive function of MDSCs in breast cancer [36]. MAPK signaling is another pathway, which we found to be significantly upregulated in tumor-infiltrating I-MDSCs from CRC patients. Studies have demonstrated that MAPK signaling pathway is essential for the activation and differentiation of myeloid cells [37, 38]. By combining these findings, we could anticipate that I-MDSCs in CRC microenvironment are activated and recruited to tumor sites where they differentiate into mature phenotypes and become immune suppressive under the influence of MAPK- and IL-6-mediated signaling pathways. Targeting these pathways in I-MDSCs could suppress their activity and reduce their accumulation within the TME, which in turn may improve the anti-tumor immune response and diminish tumor growth/metastasis.

Maintaining transcriptional homeostasis requires a dynamic equilibrium of HAT and HDAC activity, where the former favors gene transcription and the latter suppresses it [39]. In pathological conditions, including cancer, this equilibrium can be dysregulated by certain factors, causing either the silencing of tumor suppressor genes or inducing the expression of genes promoting tumor growth/metastasis and immunosuppression [40–43]. Based on functional annotation analyses, we found that 44% of HDAC- and 29% of DNA methylation-related genes along with 13% of transcriptional repressors were upregulated in tumor-infiltrating I-MDSCs. Notably, 90% of HAT-related genes were downregulated in tumor-infiltrating I-MDSCs. These data indicate that transcriptional regulation in tumor-infiltrating I-MDSCs could be controlled by DNA methylation and post-translational histone modifications, possibly, mediated by HDAC activation and the suppression of HAT activity. Such epigenetic changes can be responsible for downregulating genes involved in tumor suppression or T cell activation [44, 45]. Unlike I-MDSCs, we found that DNA methylation- and HDAC-related genes were downregulated in tumor-infiltrating PMN-MDSCs, suggesting that the regulation of gene expression in these MDSCs subsets is governed via differing epigenetic mechanisms. Therefore, we could propose a novel therapeutic approach by which specific populations of MDSCs can be targeted in CRC. Rather than endemic targeting of MDSCs, targeting specific populations of MDSCs by the use of appropriate epigenetic modifiers could be employed to design a well-targeted therapy for CRC patients.

Interestingly, we found that genes of JNK- and SNARE-mediated pathways were upregulated in tumor-infiltrating I-MDSCs, compared with APCs. These pathways could have a functional impact on I-MDSCs activity and CRC progression. Han et al. reported the upregulation of JNK pathway in tumor-infiltrating myeloid cells, from mouse liver cancer model, is crucial for promoting disease pathogenesis and progression [46]. Based on animal studies, it was suggested that specific targeting of JNK in myeloid cells could offer therapeutic benefits for patients with hepatocellular carcinoma [46]. In addition, JNK signaling pathway in myeloid cells has been associated with the production of Treg chemoattractant agents, such as CCL17 and CCL22 [47], indicating its potential role in MDSCs suppressive function. SNARE proteins have been associated with the regulation of cytokine secretion macrophages and dendritic cells, and inhibition of phagocytosis and antigen presentation [48], suggesting their potential function in I-MDSCs via similar means.

Our functional annotation and transcriptomic analyses showed that genes related to cell-cell signaling, cell proliferation, anti-apoptosis, and cellular migration were upregulated in tumor-infiltrating PMN-MDSCs. These data imply the potential of PMN-MDSCs function in CRC progression, which may involve the enhancement of tumor growth/metastasis and immune cell trafficking. Consistent with this, Ouzounova et al. reported that enrichment of lung TME with activated PMN-MDSCs was associated with increased expression of genes promoting tumor growth and metastasis [49]. Within the CRC microenvironment, we found that NF-κB- and IL-1β production-related genes were upregulated in PMN-MDSCs, compared with APCs. It has been reported that tumor-derived IL-1β enhances MDSCs infiltration, accumulation, and immunosuppressive activity within the TME, thereby promoting tumor progression [38, 50]. Another study by Tu et al. showed that IL-1β overexpression induces gastric inflammation and cancer through the activation of NF-κB, resulting in the accumulation of MDSCs within tumor sites [51]. In turn, NF-κB activation exerts an anti-inflammatory function on MDSCs, enhances MDSCs infiltration in tumor sites by regulating chemotaxis [52], and induces immunosuppression by upregulating the expression of IDO [36]. Therefore, we could suggest that the suppressive activity of tumor-infiltrating PMN-MDSCs in CRC is positively regulated by IL-1β/NF-κB signaling pathway.

Hypoxia is a common feature of solid tumors, which arise from reduced levels of oxygen and nutrient supply as a result of tumor outgrowth [53]. Hypoxia can mediate resistance to chemotherapy, promote tumor metastasis, and enhance MDSCs accumulation within TME [53]. Kumar et al. reported that the upregulation of HIF-1α enhances the immunosuppressive activity of myeloid cells and promotes the rapid conversion of MDSCs to tumor-associated macrophages (TAMs) [53] via the upregulation of iNOS and arginase [35]. In addition, HIF-1α can bind to the promotor of PD-L1, co-inhibitory receptor, and increase its expression on myeloid cells has been demonstrated [35]. In our study, the upregulation of HIF-1 pathway was more profound in I-MDSCs (immature myeloid cells), compared with PMN-MDSCs (mature myeloid cells), suggesting the potential importance of this pathway in I-MDSCs function/biology. On these grounds, we could speculate that I-MDSCs differentiate into the mature phenotype, TAMs, by upregulating HIF-1 pathway. In renal carcinoma, the upregulation of HIF-1 and constitutive activation of HIF-1 pathway occur due to a loss of tumor suppressor gene/function, von Hippel-Lindau (VHL), either by mutation or hypermethylation [54]. This subsequently results in the expression of angiogenic factors and genes promoting disease oncogenesis [54]. This is consistent with the finding that I-MDSCs regulate gene transcription by DNA methylation, which might be associated with tumor suppressor gene silencing. Employing therapeutic strategies to target HIF-1 could be beneficial in reducing the suppressive activity of MDSCs and the differentiation of TAMs, which ultimately should improve anti-tumor responses in patients. Using an ex vivo approach, we confirmed the importance of HDAC activation and IL-6 signaling pathway in regulating the expression of genes associated with MDSC suppressive function, such as ARG1, and genes related to myeloid cell chemotaxis, such as CCR2 and ITGAL.

## Conclusions

This study provides novel insights into the epigenetic mechanisms and signaling pathways regulating the transcriptional profile, and perhaps the function, of different myeloid cell subsets within the CRC TME. Furthermore, findings from this study indicate the importance of HDAC activation and IL-6 signaling pathway in regulating MDSC suppressive function and perhaps the recruitment of MDSCs to the CRC microenvironment.

## Methods

### Sample collection and storage

Tumor tissues (TT) and paired, adjacent normal tissues (NT) were obtained from 27 CRC patients who underwent surgery at Hamad Medical Corporation, Doha, Qatar. All patients included in the study were treatment-naïve prior to surgery and provided written informed consent prior to sample collection. Table 1 shows the clinical and pathological characteristics of all participating patients. All experiments were performed in accordance with relevant guidelines and regulations. This study was executed under ethical approvals from Hamad Medical Corporation, Doha, Qatar (protocol no. MRC-02-18-012) and Qatar Biomedical Research Institute, Doha, Qatar (protocol no. 2018-018). Tissue specimens were cut into small pieces and frozen in 1 ml of freezing medium (10% dimethylsulphoxide (DMSO; Sigma-Aldrich, Missouri, USA), 50% fetal calf serum (FCS; Hyclone, GE Healthcare Life Sciences, Utah, USA), and 40% RPMI-1640 medium (Life Technologies, New York, USA)), then stored in liquid nitrogen to be used in batches for subsequent analyses.

### Cell dissociation

Cells were isolated from TT by mechanical disaggregation. Briefly, tissues frozen in freezing media were thawed and washed with phosphate-buffered saline (PBS) and then mechanically cut into small pieces (~ 2–4 mm) using a surgical scalpel. Further disaggregation was performed on gentleMACS dissociator (Miltenyi Biotech, Bergisch Gladbach, Germany) without using any enzymes. The cell suspension was then passed through a 100-μM cell strainer to remove debris and aggregates. The single cell suspension was washed with PBS and stained for flow cytometric analyses and FACS sorting.

### Multi-parametric flow cytometry

Cells isolated from tissues were washed with PBS and re-suspended in 100 μl flow cytometry staining buffer (PBS with 1% FCS and 0.1% sodium azide). Fc receptors (FcR) were first blocked using FcR Blocker (Miltenyi Biotech, Bergisch Gladbach, Germany). 7-AAD viability dye (eBioscience, San Diego, USA) was used to gate live cells. Cells were then stained with cell surface antibodies against CD33-Fluorescein isothiocyanate (clone HIM3-4; BD Biosciences, Oxford, UK), CD14-phycoerythrin-Cy7 (clone M5E2; BD Biosciences), CD15-allophycocyanin (clone HI98; BioLegend, San Diego, USA), HLA DR-phycoerythrin (clone G46-6; BD Biosciences) or CD11a-phycoerythrin (clone G43-25B; BD Pharmingen, San Jose, USA) and incubated at 4 °C for 30 min. Cells were then washed twice with flow cytometry staining buffer and data were acquired by BD LSRFortessa X-20 flow cytometer (BD Biosciences).

For sorting, cells were re-suspended in Pre-Sort buffer (BD Biosciences). BD FACSAria III SORP cell sorter with BD FACSDiva software (BD Biosciences) was used. Applicable measures were taken to ensure minimal sorter-induced cell stress (SICS). Data analyses were performed on FlowJo V10 software (FlowJo, Ashland, USA).

### Cell culture

Cells were isolated from TT by mechanical disaggregation, as described above. 0.5 × 10^6^ cells per well were seeded onto a 24-well tissue culture-treated plate and cultured in complete RPMI-1640 medium (Life Technologies). Cells were untreated or pre-treated with 100 nM of HDAC inhibitor Vorinostat (SAHA, MK0683, Selleckchem, Texas, USA) or 2 μg/ml of anti-IL-6 monoclonal antibody (Sino Biological, Beijing, China) [55]. After a 2-h incubation period, 100 ng/ml of Lipopolysaccharides (LPS from *E. coli*, Sigma-Aldrich) was added to all the wells. Cells were kept in a humidified incubator at 37 °C in 5% CO_2_ for 5 days. Every other day, HDAC inhibitor and anti-IL-6 mAb were added to the wells at the same concentration. Cells were harvested on day 5 post treatment for RNA extraction.

### Library preparation

Pure 1000 CD33^+^HLA-DR^−^CD14^−^CD15^−^ (I-MDSCs), CD33^+^HLA-DR^−^CD14^−^CD15^+^ (PMN-MDSCs) and CD33^+^HLA-DR^+^CD14^+^CD15^−^ (APCs) cells were sorted from TT and NT. Cells were initially sorted from six CRC NT and TT samples. Library preparation and subsequent RNA-Seq were succeeded in subsets from two patients only (#07 and #08). This could be due to the lower percentage and lower expression level of MDSC subsets in some TT and NT samples. Similarly, CTLA-4^+^ and CTLA-4^−^ Tregs from head and neck cancer patients were compared in very small number of samples [56]. The cDNA libraries were generated using QIAseq FX Single Cell RNA Library Kit (Qiagen, Hilden, Germany) following the manufacturer’s instructions. Briefly, sorted cells were spun down and lysed immediately. The gDNA was then removed by using gDNA Wipeout buffer (Qiagen). The gDNA-removed lysates were used to generate double-stranded DNA, which were subsequently amplified using REPLI-g sc SensiPhi DNA polymerase. Small fractions of amplified products were cleaned using PureLink PCR Purification Kit (Thermo Fisher Scientific, Massachusetts, USA) and quality checked using Agilent High Sensitivity DNA Kit (Agilent Technologies, California, USA). The quality passed DNA (> 2000 bp) were quantified using Qubit dsDNA HS Assay Kit (Invitrogen, California, USA). Five hundred nanograms to 1 μg DNA was enzymatically fragmented and ligated using paired adaptors. DNA was further purified using Agencourt AMPure XP beads (Beckman Coulter, California, USA). The yield and size distribution of libraries (500–1000 bp) were determined using Qubit dsDNA HS Assay Kit (Invitrogen) and Agilent High Sensitivity DNA Kit (Invitrogen).

### RNA-sequencing data analyses

Pair end reads were quality-trimmed and were aligned to the hg19 human reference genome in CLC Genomics Workbench-12 (Qiagen) using default settings [57, 58]. The abundance of the expression of transcripts was measured as the score of TPM (transcripts per million) mapped reads in CLC Genomics Workbench 12. Abundance data were subsequently subjected to differential gene expression using built-in statistical analyses recommended in CLC Genomics protocol with 2.0-fold change and *P* value cutoff < 0.05.

### Functional annotation analyses using DAVID platform

Database for Annotation, Visualization, and Integrated Discovery (DAVID) platform was used to analyze the functional annotation, in addition to Kyoto Encyclopedia of genes and Genomes (KEGG)/BioCarta network [59, 60]. The “Functional Annotation,” “KEGG pathway,” and “BioCarta” in the online version of DAVID 6.8 was performed (https://david.ncifcrf.gov/) by matching with *Homo sapiens* gene list. The upregulated genes and downregulated genes were trimmed with both expression consistency of individual genes (> 2 FC) and *P* value cutoff < 0.05. The upregulated genes and downregulated genes were uploaded separately and focused on the Gene-Ontology, Functional-Categories, Gene-Annotations and Pathways. To visualize the network pathways, we used both KEGG and BioCarta. The related genes from functional analyses were depicted as heat plots.

### RNA extraction and reverse transcription

Total RNA was extracted from the sorted tumor-infiltrating myeloid cell populations (APCs, I-MDSCs and PMN-MDSCs) isolated from six CRC patients (#05, 07, 08, 09, 44, and 53), using RNAqueous-Micro Total RNA isolation Kit (Thermo Fisher Scientific). Total RNA was also extracted from cultured cells isolated from tumor tissue of four CRC patients (#26, 56, 60, and 63) using Total RNA Purification Plus Micro Kit (Norgen, Ontario, Canada). RNA was then amplified using 5X MessageAmp II aRNA Amplification Kit (Thermo Fisher Scientific). RNA concentrations before and after amplification were determined by Qubit RNA HS and Broad Range Assay Kit, respectively (Invitrogen). One microgram of RNA was reverse transcribed into cDNA using QuantiTect Reverse Transcription Kit (Qiagen).

### Quantitative real-time reverse transcriptase PCR (qRT-PCR)

qRT-PCR was performed using QuantStudio 6/7 Flex Real-time PCR system (Applied Biosystems, California, USA) for KAT2B, CD40, RPA4, UST, CELF2, CD38, OAS1, HDAC1, HDAC2, HDAC3, HDAC4, ARG1, IDO, ITGAL, CCR2, Wnt5a, Wnt5b, and β-actin with PowerUp SYBR Green Master Mix (Applied Biosystems). Quantification of relative gene expression was determined, using 2^−ΔΔCT^, and normalized to β-actin. Sequences for the primers are shown in Additional file 2: Table S1.

### Statistical analyses

Statistical analyses were performed using GraphPad Prism 8 software (GraphPad Software, California, USA). Paired *t* tests were performed on samples that passed the Shapiro-Wilk normality test and Mann-Whitney tests were performed for samples that did not show normal distribution. A *P* value of > 0.05 was considered statistically non-significant. The *P* values are represented as follows: ***P < 0.001, **P < 0.01, and *P < 0.05. Data are presented as mean ± standard error of the mean (SEM).

## Supplementary information

**Additional file 1: Figure S1**. Adherent junction pathway in tumor-infiltrating PMN-MDSCs. The upregulated genes in PMN-MDSCs from 2 patients were uploaded in DAVID to identify the biological pathways. Adherent junction is the top KEGG pathway regulated in PMN-MDSCs, compared with APCs within the TME. The black ovals highlight the KEGG identified functional pathways that are regulated by PMN-MDSCs, within the TME. The functional consequences of related genes are shown in red. **Figure S2**. HIF-1 signaling pathway in tumor-infiltrating I-MDSCs. The downregulated genes in PMN-MDSCs, compared with I-MDSCs from two patients were uploaded in DAVID to identify the biological pathways. HIF-1 signaling is the top KEGG pathway regulated in I-MDSCs, within the TME. The black ovals highlight the KEGG identified functional pathways that are regulated by I-MDSCs, within the TME. The functional consequences of related genes are shown in red. **Figure S3**. Validation of differential gene expression and functional network analyses of APCs, PMN-MDSCs and I-MDSCs in CRC patients. Heat maps show the TPM representing fold change to the mean expression of WNT signaling, SNARE signaling and JNK pathway activation in I-MDSCs (A). Heat maps show the TPM representing fold change relative to the mean expression of colorectal cancer-, cell migration-, NFκB-, IL-1β production-related genes in PMN-MDSCs, compared with APCs (B). Heat map shows the TPM representing fold change relative to the mean expression of tumor progression-, migration and metastasis- and DNA methylation-related genes in PMN-MDSCs **(C)**. Results obtained from four CRC patients (#09, #12, #13, and #16). **Figure S4.** CD11a expression in tumor-infiltrating PMN-MDSCs. Heat map shows the TPM representing fold change relative to the mean expression of CD11a gene (ITGAL) in PMN-MDSCs (A). Cells isolated from TT of #07 and #08 patients were stained for myeloid cell markers and CD11a, and analyzed by flow cytometry. Representative flow cytometric plots show the gating strategy employed to identify I-MDSCs and PMN-MDSCs expressing CD11a (B).

**Additional file 2: Table S1.** Gene cluster analysis.

**Additional file 3: Table S2.**: DAVID analysis

## Data Availability

The datasets used and/or analyzed during the current study are available from the corresponding author on reasonable request.

## Abbreviations

CRC: Colorectal cancer
MDSCs: Myeloid-derived suppressor cells
I-MDSCs: Immature myeloid-derived suppressor cells
IL-6: Interleukin-6
MAPK: Mitogen-activated protein kinase
M-MDSCs: Monocytic myeloid-derived suppressor cells
PMN-MDSCs: Polymorphonuclear/granulocytic myeloid-derived suppressor cells
APCs: Antigen-presenting cells
TME: Tumor microenvironment
