# Supplementary material for: Transcriptomic profiling disclosed the role of DNA methylation and histone modifications in tumor-infiltrating myeloid-derived suppressor cell subsets in colorectal cancer
Source: Clin Epigenetics. 2020 Jan 15;12:13. doi: 10.1186/s13148-020-0808-9 (PMC6964037; doi:10.1186/s13148-020-0808-9)
Supplement: Supplementary file 1 — Additional file 1: Figure S1. Adherent junction pathway in tumor-infiltrating PMN-MDSCs. The upregulated genes in PMN-MDSCs from 2 patients were uploaded in DAVID to identify the biological pathways. Adherent junction is the top KEGG pathway regulated in PMN-MDSCs, compared with APCs within the TME. The black ovals highlight the KEGG identified functional pathways that are regulated by PMN-MDSCs, within the TME. The functional consequences of related genes are shown in red. Figure S2. HIF-1 signaling pathway in tumor-infiltrating I-MDSCs. The downregulated genes in PMN-MDSCs, compared with I-MDSCs from two patients were uploaded in DAVID to identify the biological pathways. HIF-1 signaling is the top KEGG pathway regulated in I-MDSCs, within the TME. The black ovals highlight the KEGG identified functional pathways that are regulated by I-MDSCs, within the TME. The functional consequences of related genes are shown in red. Figure S3. Validation of differential gene expression and functional network analyses of APCs, PMN-MDSCs and I-MDSCs in CRC patients. Heat maps show the TPM representing fold change to the mean expression of WNT signaling, SNARE signaling and JNK pathway activation in I-MDSCs (A). Heat maps show the TPM representing fold change relative to the mean expression of colorectal cancer-, cell migration-, NFκB-, IL-1β production-related genes in PMN-MDSCs, compared with APCs (B). Heat map shows the TPM representing fold change relative to the mean expression of tumor progression-, migration and metastasis- and DNA methylation-related genes in PMN-MDSCs (C). Results obtained from four CRC patients (#09, #12, #13, and #16). Figure S4. CD11a expression in tumor-infiltrating PMN-MDSCs. Heat map shows the TPM representing fold change relative to the mean expression of CD11a gene (ITGAL) in PMN-MDSCs (A). Cells isolated from TT of #07 and #08 patients were stained for myeloid cell markers and CD11a, and analyzed by flow cytometry. Representative flow cytometri [file 13148_2020_808_MOESM1_ESM.pptx]

## Slide 1
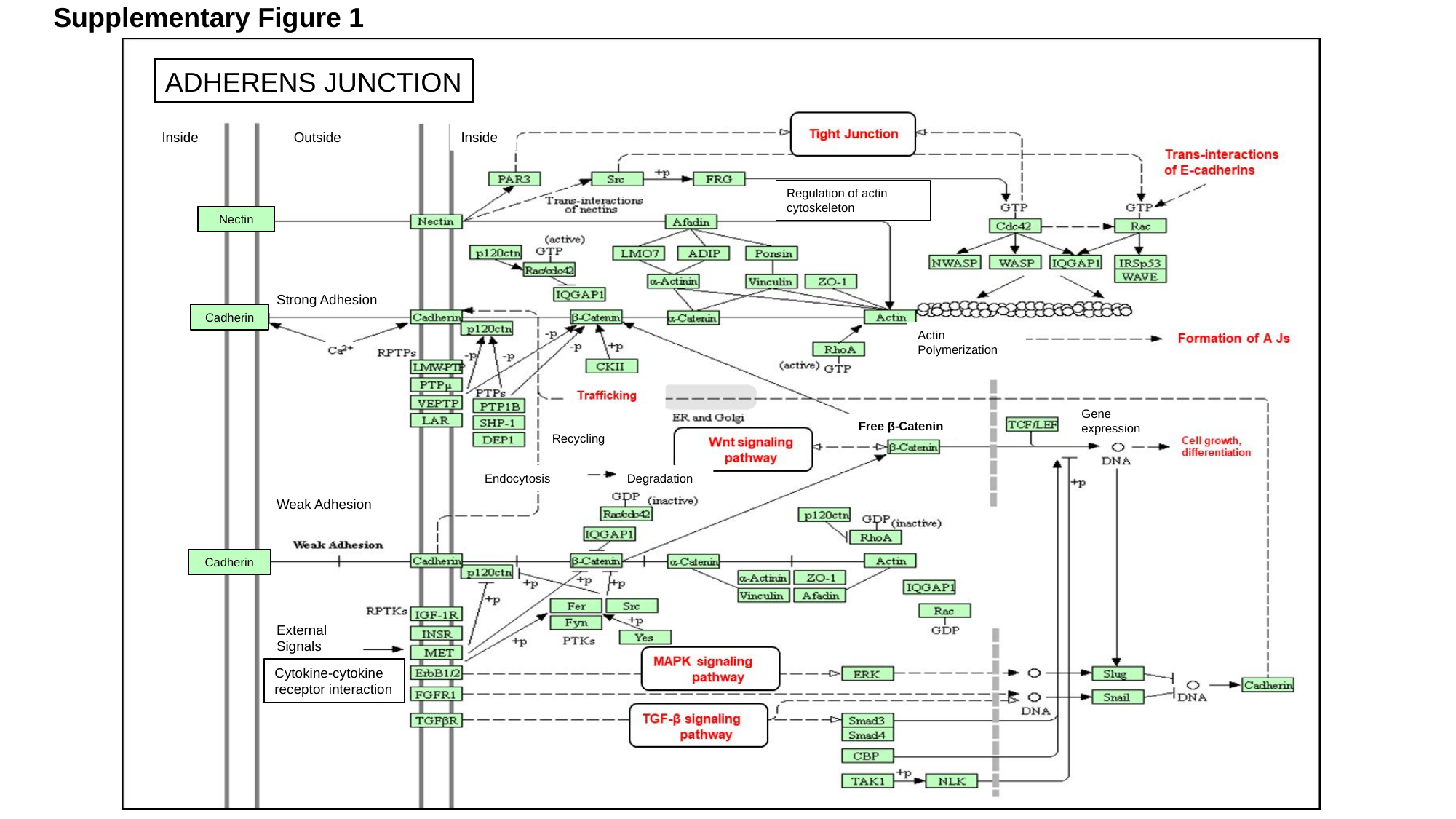

Supplementary Figure 1
ADHERENS JUNCTION
Inside
Outside
Inside
Regulation of actin cytoskeleton
Nectin
Strong Adhesion
Cadherin
Actin Polymerization
Gene
expression
Free β-Catenin
Recycling
Endocytosis
Degradation
Weak Adhesion
Cadherin
External
Signals
Cytokine-cytokine receptor interaction

## Slide 2
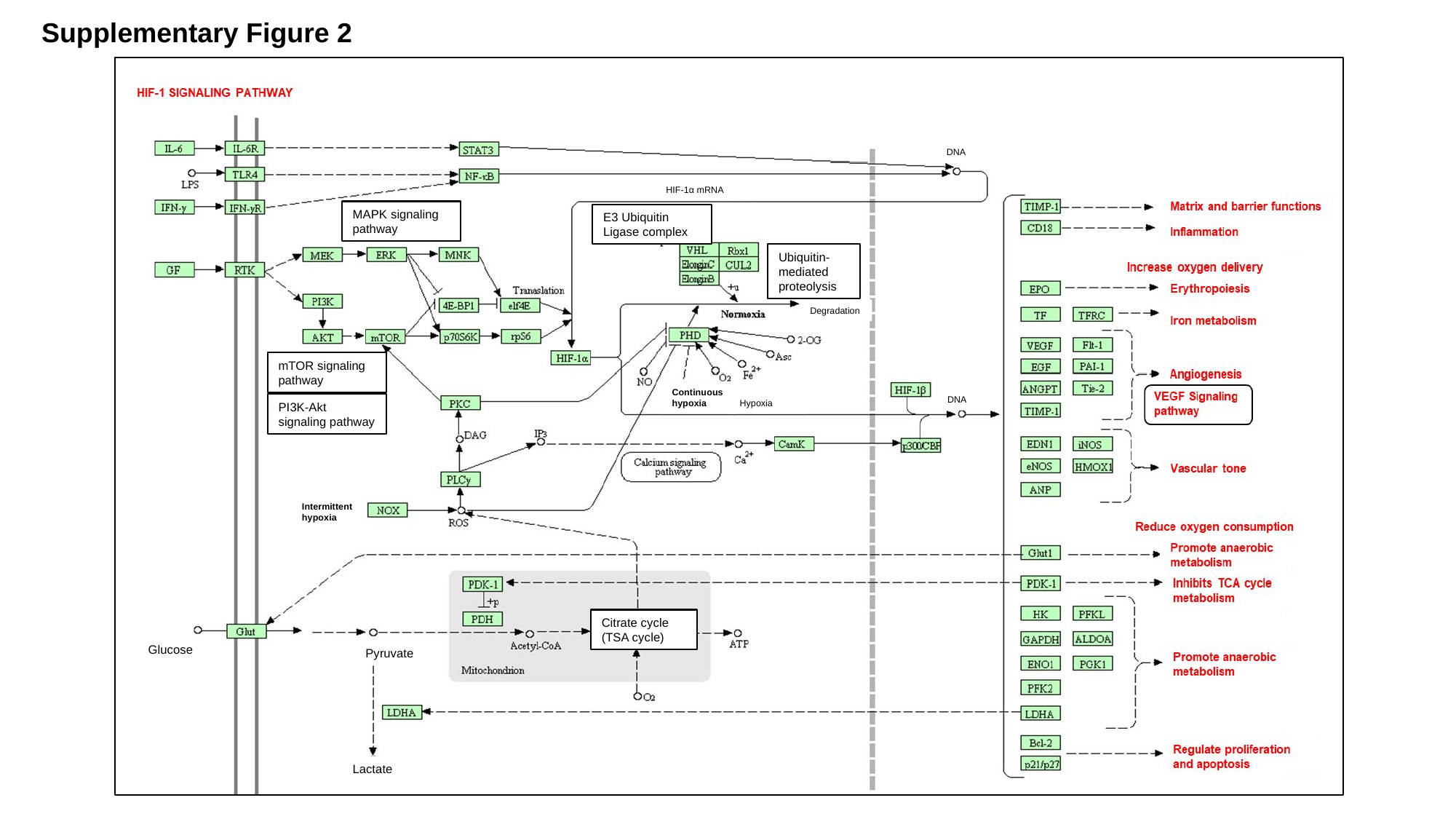

Supplementary Figure 2
DNA
HIF-1α mRNA
MAPK signaling pathway
E3 Ubiquitin Ligase complex
Ubiquitin-mediated proteolysis
Degradation
mTOR signaling pathway
Continuous
hypoxia
DNA
Hypoxia
PI3K-Akt signaling pathway
Intermittent
hypoxia
Citrate cycle (TSA cycle)
Glucose
Pyruvate
Lactate

## Slide 3
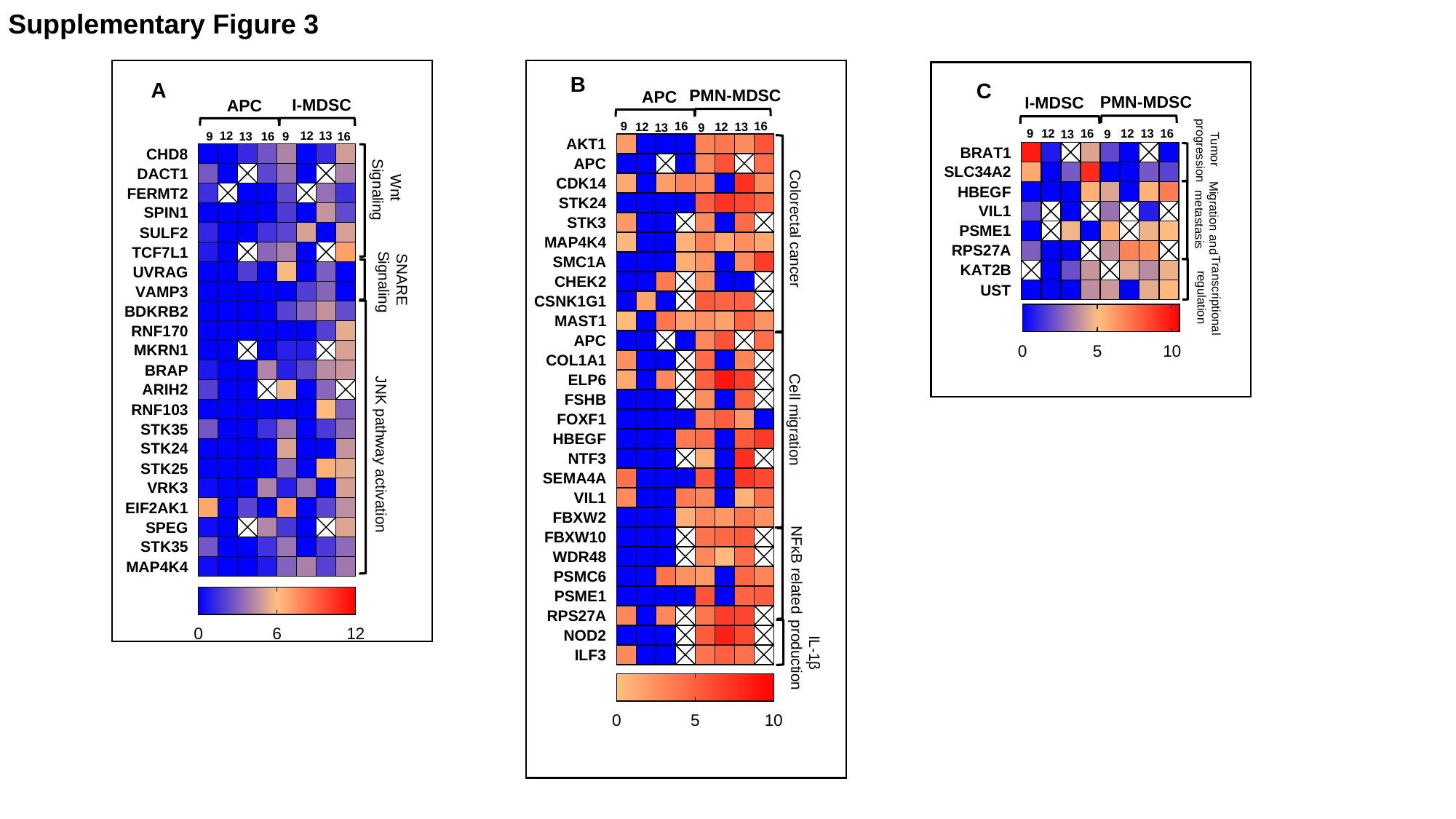

Supplementary Figure 3
B
A
C
PMN-MDSC
APC
PMN-MDSC
I-MDSC
I-MDSC
APC
16
16
9
12
12
13
13
9
16
16
9
12
12
13
13
9
12
12
13
13
9
16
16
9
Tumor
 progression
Wnt
Signaling
Migration and
metastasis
Colorectal cancer
SNARE
Signaling
Transcriptional
regulation
Cell migration
JNK pathway activation
NFκB related
IL-1β
production

## Slide 4
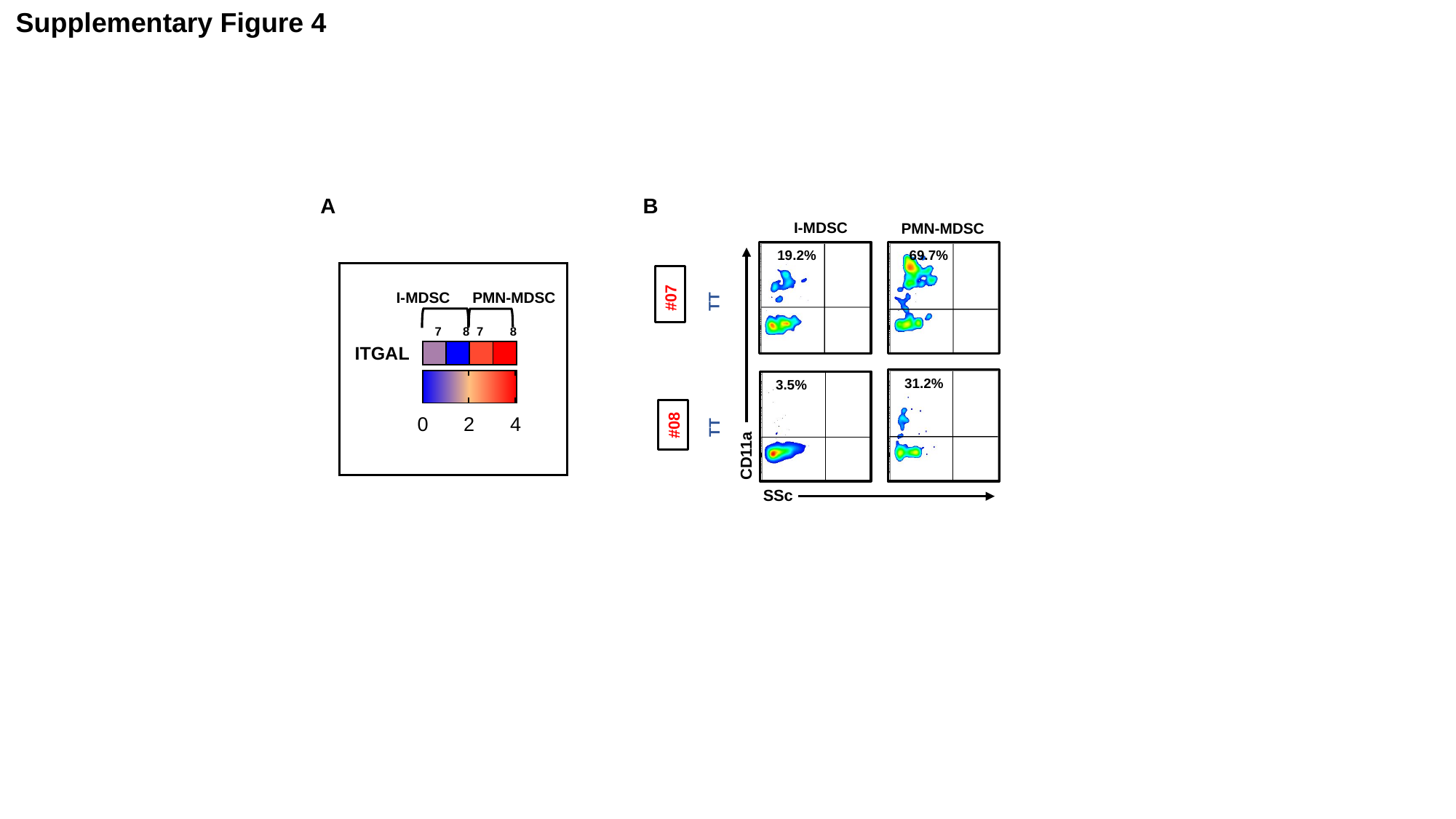

Supplementary Figure 4
A
B
I-MDSC
PMN-MDSC
19.2%
69.7%
#07
I-MDSC
PMN-MDSC
TT
7
8
7
8
31.2%
3.5%
#08
TT
CD11a
SSc
